# Supplementary material for: Lactiplantibacillus plantarum HY7718 Improves Intestinal Integrity in a DSS-Induced Ulcerative Colitis Mouse Model by Suppressing Inflammation through Modulation of the Gut Microbiota
Source: Int J Mol Sci. 2024 Jan 1;25(1):575. doi: 10.3390/ijms25010575 (PMC10779067; doi:10.3390/ijms25010575)
Supplement: Supplementary file 1 [file ijms-25-00575-s001.zip › ijms-2796861-supplementary.pdf]

Supplementary Materials: *Lactiplantibacillus plantarum* HY7718 Improves Intestinal Integrity in a DSS-Induced Ulcerative Colitis Mouse Model by Suppressing Inflammation through Modulation of the Gut Microbiota

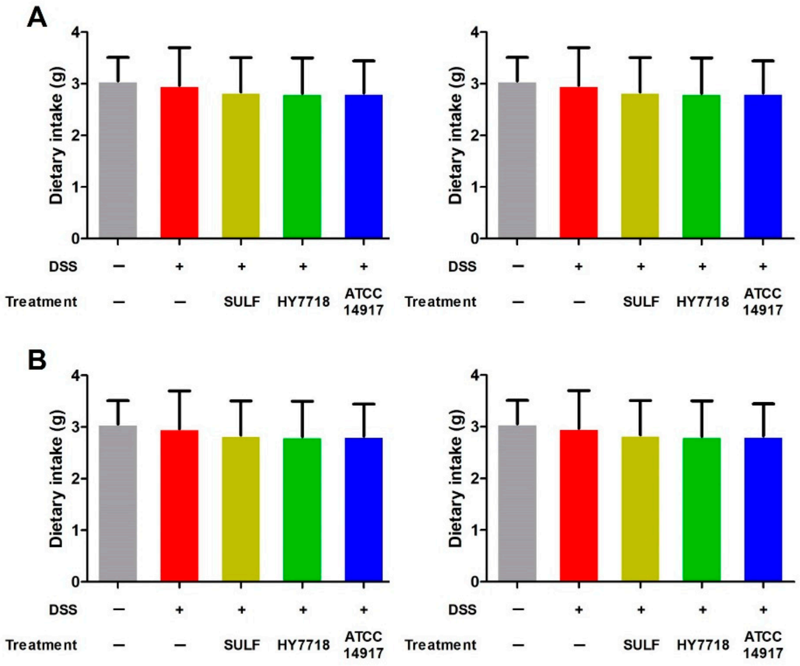

Figure S1. Dietary intake and water drinking. (A) Amounts of dietary intake (g) and water drink (mL) during animal experiments, (B) Amounts of dietary intake (g) and water drink (mL) during DSS-drinking period. Results are presented as the mean ± SD. DSS, Dextran sulfate sodium; SULF, sulfasalazine; HY7718, *Lactiplantibacillus plantarum* HY7718; ATCC 14917, *Lactiplantibacillus plantarum* ATCC 14917.

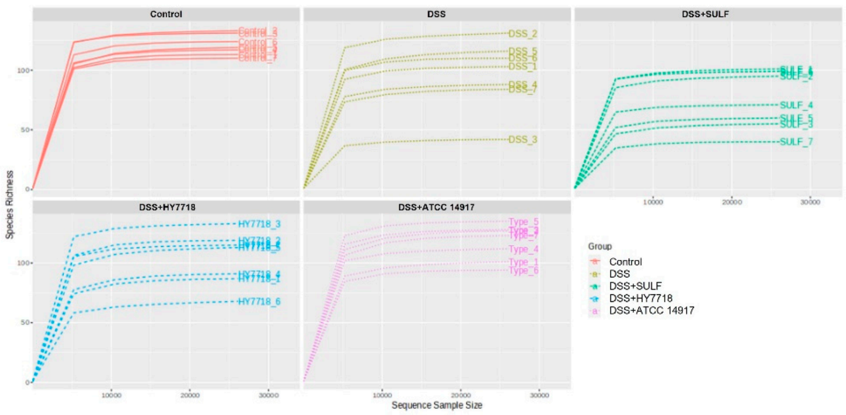

Figure S2. Rarefaction curve. DSS, Dextran sulfate sodium; SULF, sulfasalazine; HY7718, *Lactiplantibacillus plantarum* HY7718; ATCC 14917, *Lactiplantibacillus plantarum* ATCC 14917.

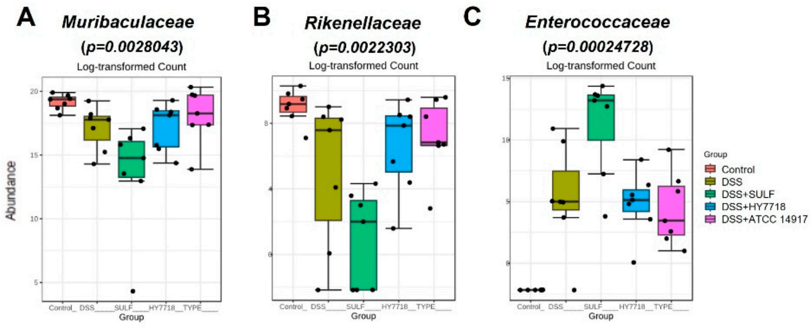

Figure S3. Relative abundance of *Muribaculaceae*, *Rikenellaceae* and *Entercoccaceae* in family level. (A) *Muribaculaceae*, (B) *Rikenellaceae* and (C) *Entercoccaceae*. Results are presented as the mean ± SD. DSS, Dextran sulfate sodium; SULF, sulfasalazine; HY7718, *Lactiplantibacillus plantarum* HY7718; ATCC 14917, *Lactiplantibacillus plantarum* ATCC 14917.
